# A new role for monomeric ParA/Soj in chromosome dynamics in *Bacillus subtilis*

**DOI:** 10.1002/mbo3.1344

**Published:** 2023-01-16

**Authors:** David M. Roberts

**Affiliations:** ^1^ School of Life Sciences University of Warwick Coventry UK

**Keywords:** *Bacillus*, microbial cell biology, molecular and bacterial genetics

## Abstract

ParABS (Soj‐Spo0J) systems were initially implicated in plasmid and chromosome segregation in bacteria. However, it is now increasingly understood that they play multiple roles in cell cycle events in *Bacillus subtilis*, and possibly other bacteria. In a recent study, monomeric forms of ParA/Soj have been implicated in regulating aspects of chromosome dynamics during *B. subtilis* sporulation. In this commentary, I will discuss the known roles of ParABS systems, explore why sporulation is a valuable model for studying these proteins, and the new insights into the role of monomeric ParA/Soj. Finally, I will touch upon some of the future work that remains.

## Graphical abstract

ParABS (Soj‐Spo0J) systems and SMC complexes are critical mediators of chromosome dynamics in a range of bacteria. In this commentary, I review the roles of these systems in *Bacillus subtilis*, as well as discuss a recent study that implicates a monomeric variant of ParA/Soj as a novel regulator of SMC release and redistribution after loading onto the DNA, a process that is critical for chromosome reorganization during axial filament formation in early sporulation.

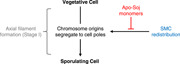

## 1 PARABS (SOJ‐SPO0J) SYSTEMS AND BACTERIAL CHROMOSOME SEGREGATION

**Figure 1:**
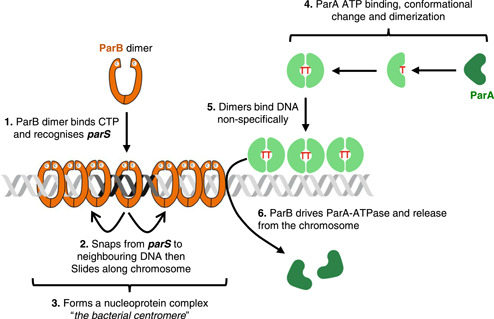
The ParABS system. The figure shows essential features of the bacterial ParABS system as well as key DNA binding steps and interactions. Protein and DNA cartoons were adapted from Roberts et al. (2022).

ParABS systems have long been implicated in plasmid and chromosome segregation in bacteria (Baxter & Funnell, 2014; Jalal & Le, 2020), and as the name suggests, are composed of three parts. The first is the DNA binding site called *parS*, which is often found in multiple copies in a region of the bacterial chromosome called the origin (or *oriC*, so named because it is where DNA replication is initiated) (Livny et al., 2007). *parS* sites are bound by dimers of the second component, the CTP hydrolase ParB, which form nucleoprotein complexes around *parS* (Figure 1, 1–3) (Spo0J in *Bacillus subtilis*) (Graham et al., 2014; Jalal et al., 2020; Murray et al., 2006; Osorio‐Valeriano et al., 2019, 2021; Soh et al., 2019). The final component of ParABS systems is the Walker ATPase ParA (Soj in *B. subtilis*). Binding of ATP to empty (or Apo)‐ParA monomers causes a conformational change that drives dimerization and nonspecific DNA binding (Figure 1, 4–5) (Leonard et al., 2005; Lim et al., 2014; Vecchiarelli et al., 2014; Zhang & Schumacher, 2017). The N‐termini of ParB interact with ParA‐ATP dimers, stimulating the latter's ATPase activity and release from the DNA (Figure 1, 6) (Leonard et al., 2005; Scholefield et al., 2011; Zhang & Schumacher, 2017).

**Figure 2:**
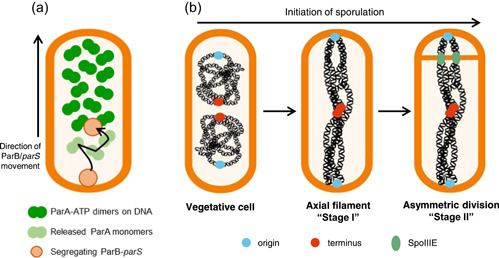
Chromosome segregation in bacteria. (a) the bacterial DNA relay/ratchet system is a model for bacterial chromosome segregation and involves ParA‐dimers (dark green circles) bound nonspecifically across the DNA (not shown). Upon DNA replication, the newly replicated ParB‐*parS* complex (orange circle) encounters nearby DNA‐bound ParA dimers, where interactions between ParA and B stimulate ParA's ATPase activity, which causes a conformational shift in ParA and its release from DNA as monomers (light green circles). ParB*‐parS* then interacts with another ParA‐dimer. By repeating this process, ParB‐*parS* migrates along the chromosome towards the opposite cell pole (as indicated by arrow), stripping off ParA dimers as it goes. Directionality is driven by a slow DNA re‐binding of released ParA. (b) As a vegetative cell (left cell) induces sporulation, the replicated chromosomes (black) reorganize to form the axial filament (middle cell), where origins (blue circles) are segregated to cell poles, and termini (red circles) remain together at mid cell. Asymmetric division (right cell) then defines the small prespore and large mother cell. The prespore chromosome becomes bisected and is bound by the DNA translocase SpoIIIE (green circles). Cell cartoons were adapted from Roberts et al. (2022).

Precisely how the ParABS (Soj‐Spo0J) system drives chromosome segregation has been subject to intensive research in a range of bacterial species (Glaser et al., 1997; Iniesta, 2014; Jalal & Le, 2020; Jung et al., 2019; Lim et al., 2014; D. C. H. Lin & Grossman, 1998; L. Lin et al., 2017). The most prominent model is called the DNA ratchet/relay system, such as that described for *Caulobacter crescentus* (Figure 2a) (Lim et al., 2014; Schofield et al., 2010; Surovtsev, Campos, et al., 2016; Surovtsev, Lim, et al., 2016; Toro et al., 2008). Similar models have been proposed in other bacterial species and for the segregation of low‐copy plasmids (Hu et al., 2015; Hwang et al., 2013; Taylor et al., 2021; Vecchiarelli et al., 2013, 2014; Wang et al., 2013).

Several critical features of the DNA relay system enable efficient chromosome segregation. The first is that upon DNA replication the majority of ParA is dimeric and bound nonspecifically across the chromosome, such that bound dimers can interact with a ParB‐*parS* complex. Second, the cycling of ParA from dimeric‐to‐free monomeric‐to‐dimeric forms requires conformational shifts. This precludes immediate rebinding of ParA to the DNA following stimulation of its ATPase activity, and so allows interaction of the ParB‐*parS* complex with the next closest ParA dimer. This provides a directionality to the movement of the ParB/Spo0J‐*parS* complex (and by extension, the chromosome).

## 2 SOJ AND SPO0J CONTROL CELL CYCLE EVENTS IN *B. SUBTILIS*

As well as being part of the ParABS system, Soj (ParA) and Spo0J (ParB) have been implicated in controlling other key aspects of the bacterial cell cycle. In *B. subtilis*, dimer and monomer forms of Soj were shown to activate or inhibit DNA replication, respectively (Murray & Errington, 2008; Scholefield et al., 2011, 2012). Additionally, Spo0J was identified as the specific loading factor for SMC complexes, which are also known as Condensin (initially in *B. subtilis* and more recently in a range of other bacteria) (Böhm et al., 2020; Gruber & Errington, 2009; Minnen et al., 2011; Sullivan et al., 2009; Tran et al., 2017). SMC/Condensin are conserved from bacteria to humans and in *B. subtilis*, are major organizers of the chromosome: following loading at the origin, SMC complexes use ATPase activity to translocate towards the terminus, meanwhile aligning the chromosome arms in a process that facilitates bulk chromosome segregation, before being specifically unloaded at the terminus (Diebold‐Durand et al., 2017; Karaboja et al., 2021; Vazquez Nunez et al., 2019; Wang et al., 2015, 2017, 2018). Finally, Soj and Spo0J have been implicated in the early stages of sporulation: by activating a checkpoint regulator, and through involvement in chromosome remodeling and segregation events (Burkholder et al., 2001; Ireton et al., 1994; Kloosterman et al., 2016; Sharpe & Errington, 1996; Veening et al., 2009; Wu & Errington, 2003).

These studies have highlighted the diverse roles that ParA/Soj and ParB/Spo0J can play in bacteria and demonstrated that these proteins have critical roles that lie beyond DNA relay‐based chromosome segregation.

## 3 SPORULATION AS A TOOL TO STUDY *B. SUBTILIS* CHROMOSOME SEGREGATION

**Figure 3:**
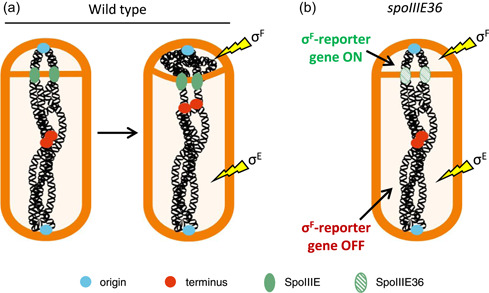
SpoIIIE, alternate sigma factors, and the trapping assay. (a) SpoIIIE (green) translocates the bisected chromosome into the small prespore. During this process, alternate sigma factors are activated in the prespore and then the mother cell (yellow lightning bolts). (b) SpoIIIE36 (hatched green circles) cannot pump the bisected chromosome. Since *σ* ^F^ and *σ* ^E^ are still activated in these cells, *σ* ^F^‐dependent reporter genes (e.g., fluorescent proteins or β‐galactosidase) can be placed on the chromosome and will be expressed if the corresponding DNA region is located within the prespore. This trapping assay has been widely used to isolate mutants involved in segregating and/or anchoring the origin regions (blue) to the pole. Cell outlines and chromosome cartoons were adapted from Roberts et al. (2022).

In times of nutrient scarcity, *B. subtilis* cells can initiate sporulation (Errington, 2003; Tan & Ramamurthi, 2014). As well as being an extremely well‐characterized developmental system that involves many of the classic steps in differentiation and development in higher organisms (asymmetric division, differential gene expression, alternate cell fates, etc.), sporulation can be broadly characterized by a series of morphological stages (Stages 0–VII), resulting in the release of a mature spore (Errington, 2010; Ryter, 1965). During early sporulation, the replicated chromosomes reorganize into an elongated structure called the axial filament (or Stage I) (Figure 2b) (Ben‐Yehuda et al., 2003; Bylund et al., 1993; Glaser et al., 1997; Ryter, 1965; Willis et al., 2020; Wu & Errington, 2003). Proper axial filament formation is critical to allow chromosome capture in the small prespore compartment after asymmetric cell division (Stage II) (Figure 2b) (Wu & Errington, 1994, 2003). In asymmetric division, a membrane protein, SpoIIIE (FtsK), binds the DNA at the point where it becomes bisected by the closing septum. This prevents the scission of the DNA before translocation of the mother‐cell localized portion of the bisected chromosome into the prespore (Figure 3a) (Bath et al., 2000; Burton et al., 2007; Fiche et al., 2013; Mohamed et al., 2021; Wu & Errington, 1997; Wu et al., 1995; Yen Shin et al., 2015). Alternate sigma factors are concomitantly activated in the prespore (*σ* ^F^) and mother cell (*σ* ^E^), which drive differential gene expression in the two compartments (Lewis et al., 1994).

A critical and widely used tool in investigating the segregation and capture of chromosome origins at the cell pole resulted from the isolation of a specific mutant of the SpoIIIE pump (called *spoIIIE36*) (Figure 3b) (Wu & Errington, 1994). *spoIIIE36* mutants divide asymmetrically and bisect the chromosome just like wild‐type cells, but the SpoIIIE36 protein is unable to translocate the bisected chromosome (Besprozvannaya et al., 2014; Wu & Errington, 1994, 1998). As a result, any DNA localized in the mother cell becomes permanently stuck in this (wrong) compartment, and yet crucially, *σ* ^F^ and *σ* ^E^ are still activated in the prespore and mother cell, respectively (Wu, 1997; Wu & Errington, 1994). As a result, the so‐called trapping assay was developed to identify genes involved in moving and/or anchoring the origin to the pole (Figure 3b) (Kloosterman et al., 2016; Lewis et al., 1994; Wagner‐Herman et al., 2012; Wu & Errington, 1998, 2003). Using this approach, mutants can be generated in a *spoIIIE36* strain, sporulation then induced, and any effect on the movement or capture of the chromosome in the prespore can be determined. As well as this, the process from inducing sporulation to asymmetric division takes 1–2 h (rather than a ~30 min doubling time for rapidly growing vegetative *B. subtilis*), and the segregating chromosome in each sporulating cell carrying the *spoIIIE36* mutation will get stuck in the translocase at the same point, effectively synchronizing the population of cells in the assay to provide homogenous readouts and a more user‐friendly timescale for experiments. This approach has therefore been widely used to identify a range of genes involved in chromosome movement and capture, including *soj* and *spo0J* (Kloosterman et al., 2016; Sullivan et al., 2009; Wagner‐Herman et al., 2012; Wu & Errington, 1998, 2003).

## 4 SOJ IS INVOLVED IN CHROMOSOME SEGREGATION DURING SPORULATION

In our recent manuscript (Roberts et al., 2022), we aimed to further characterize the role of Soj/ParA in chromosome dynamics in *B. subtilis* and presented three main advances. We showed that: (1) monomeric variants of Soj have active roles in chromosome segregation; (2) one of these monomers, Apo‐Soj, is a likely regulator of SMC complex release from *parS* sites; (3) a major redistribution of SMC complexes along the chromosome drives axial filament formation (or Stage I) during the initial stages of *B. subtilis* sporulation.

Previous studies had implicated Soj in the movement and/or anchoring of the origins at the cell pole during axial filament formation since deletions of *soj* caused the origins to fail to localize within the prespore during the trapping assay (Kloosterman et al., 2016; Wu & Errington, 2003). This “origin out” phenotype was similar to that seen in other mutants (such as deletions of *minD, minJ, sirA*, and *comN*), suggesting that Soj was operating in the same capture pathway as these factors (Duan et al., 2016; Kloosterman et al., 2016). Furthermore, there appeared to be a genetic hierarchy to this: DivIVA > ComN and MinJ > MinD > Soj, whereby loss of any component results in failure of all downstream factor(s) to localize to the pole, as well as a loss of polar origin anchoring (Kloosterman et al., 2016). Soj is at the base of this hierarchy, which is consistent with it acting at the interface between the chromosome and the cell pole (alongside Spo0J). In addition to Soj‐Spo0J and the polar complex, a second redundant pathway exists to tether origin regions to the cell pole and is based on the sporulation‐specific RacA protein (Ben‐Yehuda et al., 2003, 2005; Wu, 2002; Wu & Errington, 2003).

## 5 *B. SUBTILIS* SOJ LOCALIZES DIFFERENTLY TO PARA IN OTHER ORGANISMS

Strikingly, and in contrast with other tested ParA‐systems, fluorescent fusions to Soj do not localize nonspecifically across the chromosome, as would be expected if a DNA relay/ratchet system was operating (Murray & Errington, 2008; Roberts et al., 2022). There are two well‐characterized mutant alleles of *soj*, namely *sojG12V* (ATP‐Soj hereafter) and *sojK16A* (Apo‐Soj hereafter) that act, respectively, as ATP‐bound or empty monomers in vitro (Scholefield et al., 2011). These proteins also failed to localize across the chromosome (Roberts et al., 2022). By contrast, mutants in *B. subtilis* Soj that are known to be locked as ATP‐sandwich dimers do bind nonspecifically to the chromosome (Leonard et al., 2005; Murray & Errington, 2008). These findings suggested two things. First, ATP‐Soj and Apo‐Soj mutants are likely to be largely monomeric in vivo, and second, since the wild‐type protein localized identically to the ATP‐Soj mutant, most of the native Soj population is also predominantly in monomeric form in vivo (Roberts et al., 2022). Taken together, these findings prompted us to ask whether the DNA relay system is the driver of chromosome movement during axial filament formation, or whether Soj acts via an alternate mechanism.

## 6 TESTING THE FUNCTIONALITY OF MONOMERIC FORMS OF SOJ

As Soj may function in a monomer form in vivo, Roberts et al. (2022) conducted the trapping assay on wild‐type, ATP‐Soj, and Apo‐Soj and strikingly observed that ATP‐Soj appeared functional for chromosome segregation. This was the case even when the redundant *racA*‐based anchor system was deleted, albeit to a lesser extent. Remarkably, this result revealed, for the first time to our knowledge, that Soj does not need to dimerize, bind DNA or undergo a functional ATPase cycle to enable chromosome segregation during *B. subtilis* sporulation. In marked contrast, the origins failed to segregate and anchor to the pole in Apo‐Soj mutants (Roberts et al., 2022).

Given that the relay/ratchet system is probably not operating to drive segregation of the chromosome origins to the opposite cell poles in these sporulating cells, along with the premise that monomeric Soj cannot bind DNA, it was reasoned that another component must be bridging Soj and the chromosome (Roberts et al., 2022). There were two main candidates: SMC complexes and Spo0J and it turns out that both are involved.

## 7 A LINK BETWEEN SMC COMPLEXES AND THE AXIAL FILAMENT

To test whether SMC complexes were involved in bridging between Soj and the chromosome during axial filament formation, Roberts et al. (2022) conducted ChIP‐Seq against SMC during sporulation, which revealed that ATP‐Soj and Apo‐Soj have contrasting effects on the enrichment of SMC complexes around the origin (with more or less present, respectively). This was the first suggestion that monomeric Soj and SMC may be linked.

To interrogate this further, the localization of SMC was explored. It had been established that in vegetative cells there are 2–4 SMC foci per cell, localized at *oriC* regions where they are loaded (Gruber & Errington, 2009; Sullivan et al., 2009). This is because in these cells multiple rounds of replication can occur simultaneously. However, as cells enter sporulation, new rounds of replication are inhibited resulting in two complete copies of the chromosome in each cell (Hauser & Errington, 1995; Jameson et al., 2014; Rahn‐Lee et al., 2011; Wagner et al., 2009). Consistent with a previous study (Wang et al., 2015), Roberts et al. (2022) showed an increased number of SMC foci along the length of the axial filament, suggesting that as well as forming foci at the two polar‐localized origins, SMC becomes redistributed along the length of the axial filament. Due to the nature of SMC complexes as major organizers of chromosomes in all cells (Hirano, 2016; Yatskevich et al., 2019), it was postulated that SMC complex redistribution is a major factor in driving axial filament formation during sporulation. This was supported by seeing SMC redistribution in ATP‐Soj cells (which are functional for chromosome segregation), something which did not occur in Apo‐Soj cells (where chromosome segregation and axial filament formation fail). Altogether, these data pointed towards SMC redistribution as being a key driver of axial filament formation in a process somehow regulated by monomeric Soj.

## 8 A NOVEL POSTLOADING RELEASE REGULATOR OF SMC COMPLEXES?

**Figure 4:**
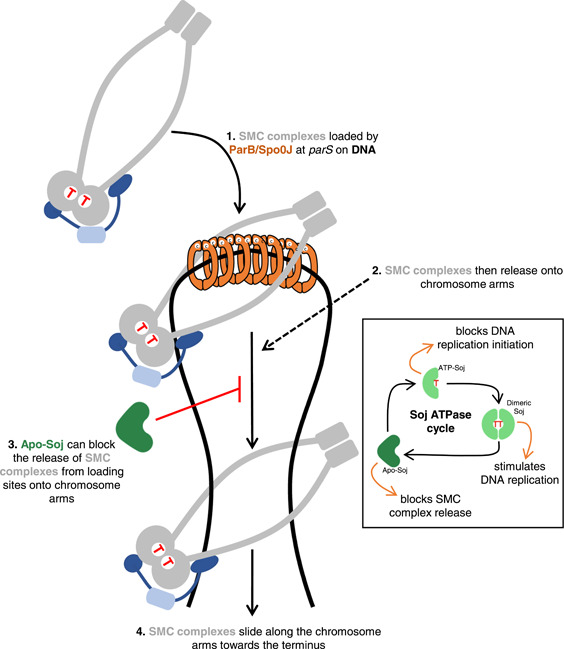
Working model for the role(s) of Soj in *B. subtilis*. Spo0J (orange), bound on DNA around *parS* sites, loads SMC complexes (gray) onto the chromosome (step 1). After loading, SMC complexes become released from Spo0J‐*parS* complexes onto the chromosome arms (step 2). Alongside SMC loading, Soj transitions through the ATPase cycle (inset box). As populations of Soj within the cell flux through the different states in this cycle, various aspects of DNA replication and chromosome segregation are regulated (indicated by orange arrows in the inset). Apo‐Soj has been newly implicated in regulating chromosome segregation by interacting with the Spo0J N‐terminus and blocking the SMC release from Spo0J‐*parS* (step 3), at least during sporulation. Upon ATP binding, ATP‐Soj negatively regulates DNA replication initiation. SMC loaded at Spo0J‐*parS* becomes released and travels along the chromosome towards the terminus, aligning and organizing the chromosome arms and driving axial filament formation during sporulation (step 4). Finally, ATP‐Soj dimerizes, binds DNA, and stimulates DNA replication initiation. The precise molecular details of the in vivo flux of the Soj forms, as well as how interactions between Soj (especially Apo‐Soj), Spo0J, and SMC regulate SMC release remain to be determined (see main text for details). Soj, Spo0J, and ATPase cycle cartoons were adapted from Roberts et al. (2022).

A reasonable initial assumption was that since cells expressing Apo‐Soj appeared to fail in chromosome segregation, the empty Soj variant probably had no function in this regard, similar to *Δsoj* (Roberts et al., 2022). However, *Δsoj* did not affect the redistribution of SMC during sporulation, in stark contrast to Apo‐Soj. This strongly suggested the latter must have a role in *B. subtilis*, albeit a negative one (Roberts et al., 2022). From a series of genetic experiments, it was subsequently shown that Apo‐Soj was dominant negative to wild type; its activity appeared to block axial filament formation and to cause a chromosome segregation delay in vegetative cells (Roberts et al., 2022). Furthermore, ChIP‐Seq revealed that SMC complexes were specifically enriched around *parS* sites in Apo‐Soj mutants. Since SMC complexes are loaded by Spo0J at *parS* sites (Gruber & Errington, 2009; Sullivan et al., 2009), these findings supported a model in which Apo‐Soj allows the loading of SMC onto the chromosome but prevents its release from loading sites onto the chromosome arms. In other words, Apo‐Soj is a likely novel regulator of SMC release from Spo0J‐*parS* (Figure 4) (Roberts et al., 2022).

How then might Apo‐Soj control the release of SMC from *parS* sites? Roberts et al., 2022 hypothesized that this was most likely to occur via interaction with Spo0J (ParB). Indeed, it has been shown that the N‐terminus of Spo0J interacts with both Soj and SMC complexes (Bock et al., 2022; Gruber & Errington, 2009). Gruber & Errington, 2009 isolated a *spo0J* mutation (*spo0JL5H*) that abolishes the interaction with Soj and therefore generates an asporogenous phenotype, but retains the ability to support SMC (Gruber & Errington, 2009). In these cells, wild‐type Soj accumulates as an ATP‐dimer, driving DNA over‐replication and blocking sporulation via the replication checkpoint (Burkholder et al., 2001; Murray & Errington, 2008; Veening et al., 2009). On combining Apo‐Soj and this *spo0J(L5H)* mutant, the negative outcome of Apo‐Soj in capturing the origin in the prespore was significantly rescued. These observations pointed to the effects of Apo‐Soj on SMC being mediated through an interaction with the Spo0J N‐terminus (Roberts et al., 2022).

## 9 SOJ, SPO0J, SMC, AND THE CELL CYCLE: NEXT STEPS

It has become clear from a range of studies, cited throughout this commentary, that Soj, Spo0J, and SMC complexes are all central players in the key cell cycle events of DNA replication, chromosome segregation, and sporulation in *B. subtilis*. In our recent paper (Roberts et al., 2022), we further expand the complex network of regulatory interactions between Soj, Spo0J, and SMC by implicating monomeric variants of Soj in the regulation of SMC complex dynamics via interactions with Spo0J, at least during sporulation. That monomeric forms of ParA/Soj are likely involved in chromosome segregation provides another layer of complexity for ParA proteins as biological switches that can integrate multiple processes (in this case chromosome segregation and replication) in a coordinated and sequential manner as ParA/Soj traverses the ATP‐cycle. As well as this, Roberts et al. (2022) provide a possible first example of a regulatory step in controlling SMC movement on the chromosome after the initial loading event mediated by ParB/Spo0J (Figure 4).

Two critical next steps will be to structurally confirm that *B. subtilis* Apo‐Soj and ATP‐Soj monomers have alternate structures (as has been proposed for ParA from other species) (Vecchiarelli et al., 2010), as well as to biochemically confirm that Soj is a monomer in vivo. For the latter, one approach could be to use site‐specific crosslinking, for example via cysteines introduced into the dimer interface. This approach will likely be technically challenging due to the need to avoid inadvertently monomerizing Soj when introducing the cross‐linkable residues (e.g., in the dimerization interface). However, alternate crosslinking approaches with reagents such as formaldehyde are unlikely to be specific enough, because capturing “near neighbor” interactions will not distinguish between genuine Soj dimers and monomeric variants that are next to each other in the complex with the Spo0J arrays at the origin.

Another outstanding question concerns precisely how monomeric Soj variants (particularly Apo‐Soj) mediate their effect on SMC release from *parS*, which appears to occur via Spo0J (Roberts et al., 2022). One hypothesis is that upon interacting with the Spo0J N‐terminus, Apo‐Soj may somehow affect the Spo0J CTP cycle. Identifying the SMC distribution at *parS* sites in point mutants of Spo0J blocked throughout its nucleotide cycle (alongside Apo‐Soj) may help elucidate this further (Antar et al., 2021; Osorio‐Valeriano et al., 2021).

Third, it will be interesting to establish how conserved the role of monomeric Soj/ParA (particularly the Apo variant) is in regulating aspects of the cell cycle in other bacterial species. A recent preprint has suggested that Apo‐ParA in *Caulobacter crescentus* was dominant lethal, and when an Apo‐ParA‐mCherry fusion (which could grow) was expressed alongside wild‐type ParA, cells displayed a minicell phenotype (Menikpurage et al., 2022). It thus appears that monomeric variants of Soj can have specific functional roles in an alternative bacterial species, underscoring the need to test the role of these variants in other established model and nonmodel systems.

An intriguing aspect of Soj in *B. subtilis* is its role at the cell pole. It could be that polar Soj is simply a manifestation of the need to anchor origins at the pole during sporulation. However, since it also localizes to the pole during vegetative growth, it remains possible that there is another role for this population of Soj—for example, to provide a dimerization hub for ATP‐Soj away from the origin. It is known that recruitment of Soj to the cell pole is dependent on MinD (another ParA‐like Walker ATPase involved in cell division control) (Autret et al., 2001; Kloosterman et al., 2016; Murray & Errington, 2008), but the exact molecular basis of the interaction, and its function, will be some of the key next steps in understanding Soj dynamics.

## 10 CONCLUSION

Soj has primary roles in regulating multiple critical aspects of DNA replication and chromosome segregation, and it is emerging that this control can be exerted as Soj transitions through its ATP cycle (including in both its monomeric and dimeric forms). In the model Gram‐positive bacterium *B. subtilis*, deciphering these processes has been greatly aided by exploiting the highly characterized and genetically tractable system of sporulation. Yet despite recent advances in understanding the multiple roles of Soj in *B. subtilis* as outlined above, it is clear that there is still much more to do, specifically in understanding the precise interplay between Soj structure and function, and whether this applies to a wider range of bacterial species.

## AUTHOR CONTRIBUTIONS

**David M. Roberts**: Conceptualization (equal); writing – original draft (equal); Writing – review & editing (equal).

## CONFLICT OF INTEREST

None declared.

## ETHICS STATEMENT

None required.

## Data Availability

Not applicable.
